# Exploring Cannabidiol’s Role in Regenerative Medicine: Focus on Neural and Skeletal Tissues

**DOI:** 10.3390/biomedicines13102490

**Published:** 2025-10-13

**Authors:** Rogerio Leone Buchaim, Livia Cristina Dias, Fabiana Gomes Cardoso Pereira de Sousa, Samuel de Sousa Morais, Alexandre José Jacintho, Marina Ribeiro Paulini, João Paulo Mardegan Issa, Daniela Vieira Buchaim

**Affiliations:** 1 Department of Biological Sciences, Bauru School of Dentistry (FOB/USP), University of Sao Paulo, Bauru 17012-901, SP, Brazil; rogerio@fob.usp.br (R.L.B.); liviacdias@usp.br (L.C.D.); fabianasousa@usp.br (F.G.C.P.d.S.); 2 Graduate Program in Anatomy of Domestic and Wild Animals, Faculty of Veterinary Medicine and Animal Science, University of Sao Paulo (FMVZ/USP), Sao Paulo 05508-270, SP, Brazil; 3 Center for the Study of Venoms and Venomous Animals (CEVAP), Sao Paulo State University (UNESP), Botucatu 18610-307, SP, Brazil; 4 Medical School, University Center of Adamantina (FAI), Adamantina 17800-000, SP, Brazil; 83223@fai.com.br (S.d.S.M.); 19823@fai.com.br (A.J.J.); 5 Department of Basic and Oral Biology, School of Dentistry of Ribeirao Preto, University of Sao Paulo (FORP-USP), Ribeirao Preto 14040-904, SP, Brazil; marina.paulini@usp.br (M.R.P.); jpmissa@forp.usp.br (J.P.M.I.); 6 Postgraduate Department, Dentistry School, Faculty of the Midwest Paulista (FACOP), Piratininga 17499-010, SP, Brazil

**Keywords:** cannabidiol, nerve regeneration, bone regeneration, aging, inflammation, human endocannabinoid system, peripheral nervous system, central nervous system

## Abstract

Cannabidiol (CBD) is a non-psychotropic compound found in plants of the Cannabis genus, extensively studied for its therapeutic potential. Research has shown that CBD possesses anti-inflammatory, antioxidant, and regenerative properties, and may contribute to the recovery of neural and bone tissues. In light of the aging population and the resulting rise in neurodegenerative and osteodegenerative conditions, exploring novel therapeutic strategies that promote cellular regeneration is increasingly important. This review aims to compile and critically analyze key studies published in recent decades regarding the effects of CBD on the regeneration of the central and peripheral nervous systems, as well as bone tissue. Findings from in vivo studies indicate that CBD can attenuate inflammatory responses, inhibit oxidative stress, and modulate cellular pathways involved in tissue repair, thereby supporting neuronal and bone regeneration. Moreover, evidence suggests that CBD may protect cells from structural damage, enhancing the functional recovery of affected tissues. Despite scientific advances highlighting cannabidiol as a promising agent for bone and nerve regeneration, its therapeutic application still faces significant limitations. The primary challenge lies in the lack of robust clinical trials in humans, as most existing evidence is derived from in vitro and in vivo studies, making it difficult to confirm its efficacy and safety in clinical contexts. Additionally, CBD’s low bioavailability—due to first-pass hepatic metabolism—hinders dose standardization and reduces the predictability of therapeutic outcomes. Compounding these issues are regulatory constraints and the persistent social stigma surrounding cannabis-derived compounds, which further impede their integration and acceptance in regenerative medicine. Therefore, future research is essential to validate the therapeutic benefits of CBD and to establish its clinical applicability in treating neurological and bone disorders.

## 1. Introduction

Cannabidiol (CBD) is the most abundant non-psychoactive compound found in the *Cannabis sativa* plant [1]. It has been extensively investigated for its diverse pharmacological properties, including anxiolytic, anti-inflammatory, antioxidant, neuroprotective, chemotactic, angiogenic, anticancer, osteogenic, and analgesic effects, all of which show significant potential in the treatment of neurodegenerative and bone-related conditions such as Parkinson’s disease and Alzheimer’s disease [2,3,4,5,6]. Additionally, CBD may play a role in the regeneration of cells affected by traumatic injury or functional impairment [7].

In recent decades, scientific interest in cannabidiol has grown substantially, as reflected in the steady increase in academic publications since the mid-2000s and a peak in citations between 2019 and 2021 [8]. Concurrently, regulatory changes in various countries have facilitated greater access to the compound, thereby encouraging research into its mechanisms of action, efficacy, and safety profile. Beyond its well-established applications in neurological, psychiatric, and pain-related conditions, emerging evidence has underscored CBD’s potential in promoting bone repair and neural regeneration, further expanding its therapeutic scope [9]. As a result, continuous updates to the scientific literature are essential to advance research on this compound with such diverse and promising potential. This will help pave the way for its safe and effective clinical use, both as an adjuvant therapy and as a possible alternative to conventional treatments with addictive potential, such as opioids [10].

In this context, CBD emerges as a dual-action therapeutic agent, capable of promoting both neural and bone regeneration in an integrated manner. Within the nervous system, it supports neuroplasticity, protects against oxidative stress, and facilitates the formation of new synapses, thereby aiding in the recovery of damaged tissues. In bone tissue, CBD modulates the inflammatory response, recruits mesenchymal stem cells, and stimulates mineralization, accelerating fracture healing and enhancing the quality of newly formed bone. This dual applicability positions cannabidiol as an innovative and promising molecule in the field of regenerative medicine, particularly for conditions associated with aging, trauma, and degenerative diseases [11,12].

The study of the human endocannabinoid system (ECS) provides valuable insights into the mechanisms by which cannabidiol exerts its effects on both neural and bone tissues. This system, which plays a critical role in maintaining physiological homeostasis, comprises a network of cannabinoid neurotransmitters, receptors, endogenous agonists, and associated biochemical components distributed throughout various body structures [13]. It is involved in regulating key physiological processes such as sleep, mood, appetite, and pain perception [14,15,16]. CBD interacts with the ECS in a complex manner, primarily through indirect binding to two major G protein-coupled cannabinoid receptors: cannabinoid receptor type 1 (CB1), predominantly located in the central nervous system (CNS) and associated with cognitive functions, memory, and emotional regulation; and cannabinoid receptor type 2 (CB2), more prevalent in the peripheral nervous system (PNS), where it plays a crucial role in modulating immune responses. CBD’s interaction with cannabinoid receptors influences a range of physiological and therapeutic processes, including pain modulation, inflammation control, and the enhancement of neuroplasticity and cellular regeneration [17,18]. Through these interactions, neurotransmitters bind to their respective receptors in target tissues, contributing to functions such as cognition regulation, appetite control, and the treatment of inflammation, diabetes, and neurodegenerative diseases [19]. Studies have shown that CBD modulates key biochemical pathways, notably by attracting mesenchymal stem cells (MSCs) and targeting sites of neuronal injury through the regulation of inflammatory responses. This includes reducing levels of pro-inflammatory cytokines such as TNF-α and IL-6, increasing anti-inflammatory IL-10, stimulating chemotactic factors like SDF-1 and CXCR4, and promoting the release of vascular endothelial growth factor (VEGF) [7,20]. Once recruited, MSCs secrete neurotrophic factors such as brain-derived neurotrophic factor (BDNF) and nerve growth factor (NGF), facilitate differentiation into neural precursors, and modulate inflammation—collectively supporting neuronal regeneration [21].

The mechanisms by which CBD alleviates neuropathic pain involve the modulation of pain receptors and inflammatory pathways. One key interaction is with the transient receptor potential vanilloid type 1 (TRPV1), which CBD activates to reduce hypersensitivity in affected nerves [6]. Another mechanism involves modulation through the endocannabinoid system, which regulates pain perception. CBD promotes analgesic signaling by inhibiting the enzyme fatty acid amide hydrolase (FAAH), responsible for the degradation of anandamide, thereby enhancing its levels and prolonging its effects [6]. Additionally, CBD’s anti-inflammatory properties contribute to neuropathic pain relief by reducing the production of pro-inflammatory cytokines and decreasing glial cell activation in the nervous system [22]. Its antioxidant effects further support neuronal protection by neutralizing free radicals, which are commonly elevated in cases of nerve injury [17,18].

CBD’s osteoinductive potential is closely linked to a series of physiological events that occur during the bone healing and regeneration process, traditionally divided into three main phases: inflammation, repair, and remodeling [23].

During the inflammatory phase, a hematoma rich in immune cells forms at the injury site, creating a microenvironment conducive to the recruitment of mesenchymal stem cells and osteogenic progenitors. This is followed by the repair phase, in which a soft callus composed of fibrocartilaginous tissue begins to develop. Within this callus, chondrocytes undergo hypertrophy and progressive mineralization, facilitating the replacement of cartilage with primary bone tissue. Finally, in the remodeling phase, immature bone is gradually replaced by lamellar bone, which is structurally stronger and functionally optimized [23].

Preclinical studies suggest that CBD positively influences multiple phases of the bone repair process, from the initial inflammatory response to the final remodeling of bone tissue. During callus formation, CBD promotes the recruitment of mesenchymal stem cells and supports osteogenic differentiation by modulating inflammatory cytokine activity and stimulating factors involved in angiogenesis and mineralization. These mechanisms contribute to accelerated fracture healing and improved tissue quality, as demonstrated in animal models of femoral injury, where CBD was shown to reduce cancellous bone loss and enhance the mechanical properties of regenerated bone. Furthermore, in vitro studies using stem cells derived from dental pulp and bone marrow reinforce CBD’s osteoinductive potential, highlighting its promise as a therapeutic alternative for complex fractures, osteoporosis, and degenerative skeletal disorders. These findings position cannabidiol as an innovative and potentially less invasive option compared to conventional therapies. However, clinical validation remains essential to confirm its efficacy and safety for routine medical use [5].

**Figure 1:**
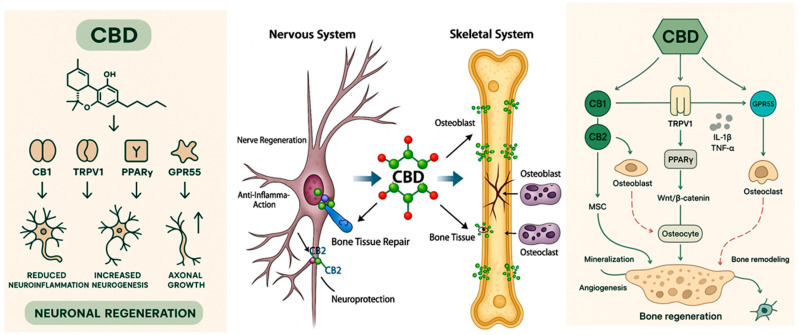
Representative image illustrating the main biological effects of cannabidiol (CBD) on the nervous system and skeletal system, highlighting its biological and therapeutic mechanisms. CBD interacts with multiple molecular targets, including CB1 and CB2 cannabinoid receptors, TRPV1 channels, PPARγ, and GPR55 receptors. Activation of CB2 promotes mesenchymal stem cell (MSC) differentiation into osteoblasts, enhancing mineralization and angiogenesis. Through TRPV1 and PPARγ, CBD modulates the Wnt/β-catenin pathway, supporting osteocyte function and bone homeostasis. Additionally, CBD reduces pro-inflammatory cytokines (IL-1β, TNF-α) and regulates osteoclast activity via GPR55, contributing to controlled bone remodeling. Collectively, these mechanisms result in enhanced osteogenesis and bone regeneration.

Therefore, the objective of this literature review is to synthesize and analyze the available evidence regarding the neuroregenerative and osteoregenerative effects associated with cannabidiol-based therapies. This analysis is particularly relevant in light of the growing aging population, which increasingly presents disorders involving neural and bone tissue degeneration [24,25]. Accordingly, it is essential to explore therapeutic strategies that not only extend life expectancy but also enhance the quality of life for affected individuals (Figure 1).

The PubMed/Medline database was primarily used to select the studies included in this review, with the aim of identifying and synthesizing the most relevant information on the topic. Eligible sources included original research articles and systematic reviews specifically focused on cannabidiol, published in English or Portuguese, and available in full text.

## 2. Nerve and Bone Diseases Associated with Aging

Simultaneously with the advancement of medicine, there has been an increase in the population’s life expectancy, and with that, an increase in the number of cases of diseases where aging is a risk factor [25,26,27,28]. Thus, the study of substances with effects capable of delaying the adverse effects of the natural aging process has become increasingly necessary [26,28].

The degeneration and death of neurons is an inherent process of aging. Despite this, dysfunctions in the functioning of these cells, which are so important for the body, directly impair the psychomotor abilities of individuals who have any condition that catalyzes the process of degradation or neuronal cell death [27]. Therefore, it is essential for maintaining the quality of life in the elderly to seek therapies that delay neuronal degradation and death, in order to preserve the structure and function of these cells [24].

The main diseases that affect the homeostasis of neurobiological functions have aging as the primary risk factor, ranging from peripheral neuropathies to neurodegenerative diseases such as Alzheimer’s, Parkinson’s, and Multiple Sclerosis [27,29,30]. Therefore, the use of drugs with effects capable of delaying the adverse effects of aging, such as CBD, has been gaining attention in the treatment of numerous conditions affecting nervous tissue, promoting its modulation and regeneration, yielding promising results in studies conducted through the observation of the effects of cannabinoid medications on the human body [29].

This fact also applies to bone regeneration. Aging is one of the factors that can interfere not only with neurological functions but also with bone homeostasis, considering that all human beings go through the process of aging [31].

Physiological aging leads to an imbalance in the activities essential for maintaining the human skeleton [32]. With age, there is a decline in the production of osteoblasts and an increase in apoptosis among them, leading to a decrease in bone mass, which can also increase the risk of fractures in the elderly, as well as the difficulty in healing such injuries [25].

Thus, due to the growing aging population, therapies that offer benefits to the quality of life of the elderly are being researched [33]. Cannabidiol is one of the potential candidates for anti-aging therapies, due to its antioxidant and anti-inflammatory properties [34]. As reported in a study using rats, cannabidiol was suggested as a good therapy option for the treatment of osteoarthritis, as the administration of the drug reduced the acute inflammatory process in the rodents, in addition to helping control pain [35].

Another disease that has been drawing attention in medicine is osteoporosis, which currently affects around 200 million people who suffer from pain and bone loss, making bone fractures more likely and potentially leading to premature death in the elderly [36,37,38]. The pharmacotherapies investigated for this type of chronic disease are anti-resorptive medications. These inhibit osteoclastic activity, making it more difficult for the bone to be reabsorbed, Similarly to these drugs, cannabidiol can not only reduce osteoclastic activity but also promote greater bone regeneration through the differentiation of osteoblasts [23,39].

Cannabidiol also stands out in dental studies, linking its regenerative and anti-inflammatory properties with diseases affecting the dental pulp, such as pulpitis (bacterial inflammation of the dental tissue). A study on this topic demonstrated that the application of cannabidiol at the site of inflammation promotes the differentiation of dental pulp cells and reduces the expression of TNF-α, which is responsible for hindering the healing process of the pulp injury [40].

The main challenge for the use of cannabidiol in the treatment of aging-related diseases is the lack of robust clinical trials that define safe doses, appropriate routes of administration, and potential adverse effects in this population. Elderly patients, for the most part, are polypharmacy patients, which increases the risk of unexpected drug interactions. This scenario is aggravated by the natural decline in liver function with age, considering that CBD is primarily metabolized in the liver. Evidence already indicates, for example, that CBD can interact with central nervous system depressants, resulting in cardiac toxicity, addictive hypertension, and tachycardia. Therefore, studies focused on CBD administration, its mechanism of action, and its safety profile in neurodegenerative and osteodegenerative diseases are essential [41,42]. Despite the risks, CBD appears to be a promising agent, especially for elderly patients, who can benefit from its potential palliative effects. However, these benefits can only be fully explored when there is more scientific evidence to reduce the likelihood of adverse events [43]. Short-term treatment with cannabis-based medical products, predominantly containing CBD or delta-9-tetrahydrocannabinol THC, resulted in a reduction in a bone resorption marker. Although this reduction was not clinically significant, the finding may indicate potential protective properties of cannabinoids on bone health. Further research with longer dosing durations is needed, particularly in individuals with specific bone conditions, such as osteoporosis. Cannabidiol (CBD) also exhibits potential therapeutic effects across a wide spectrum of neuropsychiatric disorders. Evidence suggests that CBD mitigates neural damage associated with neurodegenerative and ischemic conditions. Additionally, it demonstrates efficacy in reducing behaviors analogous to psychosis, anxiety, and depression. CBD also modulates synaptic plasticity and promotes neurogenesis. Although the precise mechanisms underlying these effects remain incompletely understood, they appear to involve multiple pharmacological targets and signaling pathways.

Clearly, the osteoregenerative, neuroregenerative, anti-inflammatory, and pain-controlling potentials of cannabidiol are already being widely researched and proven. Therefore, if used correctly and continuously studied, this medication can certainly promote an improvement in the well-being of the elderly and individuals with various health conditions.

## 3. Molecular Mechanisms of Cannabidiol

Cannabidiol acts on several cellular and physiological processes, such as inflammation [4], apoptosis [44], oxidative stress [45] and tissue regeneration [12,46]. Its effects are mediated by multiple molecular targets that contribute to the restoration of homeostasis in injured tissues, especially in the context of bone and nerve regeneration [4,47].

Cannabidiol is a phytocannabinoid with a terpene–phenolic structure and molecular formula C_21_H_30_O_2_. It is a low molecular weight molecule with a lipophilic nature, characteristics that favor its diffusion in adipose tissue and highly vascularized areas. Understanding its pharmacokinetics is essential, since the route of administration and dosage directly influence absorption, distribution, metabolism, and excretion [48,49].

Cannabidiol absorption generally peaks in plasma between 2.5 and 5 h after administration. Because it is a lipophilic substance, its bioavailability increases considerably when ingested with high-fat meals. After absorption, CBD and its metabolites exhibit high plasma protein binding. Metabolism occurs predominantly in the liver, and excretion occurs primarily in the feces [41,50].

To date, there is no established ideal dosage for CBD in regenerative therapies (bone or nerve). Therefore, the doses used follow protocols already approved by the Food and Drug Administration (FDA). Oral administration is the most common route, via oils, capsules, and other formulations, due to its convenience and greater dosage accuracy. Studies show that, when administered orally in a single dose, CBD has a plasma half-life of approximately 4 to 5 h. However, its bioavailability is relatively low, around 6%, due to extensive first-pass hepatic metabolism [51,52]. The compound is predominantly eliminated via the fecal route. In clinical practice, gradual dose titration is recommended, starting with small doses and progressively increasing them according to the patient’s response. This method is widely used in prescribing CBD for conditions such as Lennox–Gastaut syndrome, Dravet syndrome, and Tuberous Sclerosis Complex [12,53,54].

In the human body, the endocannabinoid system constitutes a neuromodulatory and homeostatic signaling network, formed primarily by cannabinoid type 1 and type 2 receptors, in addition to their endogenous lipid agonists, such as arachidonoylethanolamide (anandamide), 2-arachidonoylglycerol (2-AG), and 2-arachidonylglyceryl ether (noladin ether). Although cannabidiol does not have significant affinity to act as a direct ligand for CB1 or CB2, its pharmacological action involves indirect modulation of these receptors, as well as interaction with multiple cellular signaling pathways. Evidence suggests that CBD exerts effects on ion channels and membrane receptors, including TRPV1 (vanilloid receptor type 1), GPR55 (G protein-coupled receptor), PPAR-γ (peroxisome proliferator-activated receptor), and serotonergic receptors such as 5-HT1A. Through this diverse range of interactions, CBD participates in the regulation of metabolic, neuroprotective, anxiolytic, and anti-inflammatory processes, standing out as a multifunctional modulator of the endocannabinoid system and other associated physiological systems [43,55,56].

In addition to its indirect action on classical cannabinoid receptors (CB1 and CB2) and on regulatory enzymes of the endocannabinoid system, cannabidiol also exhibits significant modulatory activity on transient receptor potential (TRP) cation channels. These membrane receptors, widely expressed in peripheral sensory neurons, participate in the transduction of nociceptive and inflammatory signals, exhibiting an initial excitatory phase followed by a refractory state of desensitization. CBD acts on multiple TRP subtypes, including TRPV1, TRPV2, TRPV3, TRPV4, and TRPA1, while exerting an antagonistic effect on TRPM8 (mucolipin). Notable among these mechanisms is the modulation of TRPV1, a channel closely associated with inflammatory hyperalgesia. Activation of this receptor by CBD induces its subsequent desensitization, resulting in reduced neuronal excitability. Thus, the interaction of CBD with TRP channels complements its action on the endocannabinoid system, enhancing its analgesic, anti-inflammatory, and neuroprotective effects [55].

Another relevant target is the nuclear receptors PPARγ (Peroxisome Proliferator-Activated Receptors gamma), which play a central role in suppressing inflammatory responses. Activation of these receptors by CBD leads to increased production of anti-inflammatory cytokines and inhibition of inducible nitric oxide synthase (iNOS) expression in immune cells, especially endothelial cells—the first cells activated in the inflammatory process. This reduces monocyte adhesion and transendothelial migration, events that sustain chronic inflammation. Consequently, there is a decrease in pro-inflammatory cytokines such as TNF-α and IFN-γ. Furthermore, this pathway is also related to the control of cell apoptosis, promoting the elimination of damaged cells without activating additional inflammatory processes [56,57,58]

CBD also has significant antioxidant properties. During inflammation, inflammatory cells produce reactive oxygen species (ROS), which can result in DNA damage and cellular dysfunction. CBD’s antioxidant properties neutralize these reactive species, reducing oxidative stress and protecting cells from inflammatory-induced damage [4].

Structure–activity relationship (SAR) studies involving phytocannabinoids, particularly cannabidiol, have shown promise in elucidating molecular mechanisms that are not yet fully understood. Recent research demonstrates that structural manipulation of CBD derivatives can modify their pharmacological profile, increasing, for example, their affinity for the CB2 receptor. This finding is particularly relevant, since CB2 receptors are predominantly associated with the peripheral nervous system and the immune system, playing a fundamental role in modulating inflammatory and neuroimmune processes. Therefore, structural optimization of CBD may represent a significant advance in the development of therapeutic strategies aimed at treating neurodegenerative diseases and chronic inflammatory conditions [55,59].

In the nervous system, CBD interferes with chronic pain transduction by modulating sensory nociceptors, altering intracellular calcium concentrations and the resting potential of excitatory cells. This contributes not only to its analgesic effect but also to its anxiolytic effects. Additionally, through indirect mechanisms, CBD increases the concentration and effects of neurotransmitters and modulators such as endocannabinoids, adenosine, and GABA, which reinforces its anxiolytic, anticonvulsant, and neuroprotective effects [56,60,61].

In the skeletal system, CBD influences the production of bone progenitors and modulates the molecular mechanisms involved. Thus, at different stages of bone regeneration, cytokines with inflammatory and immunological functions, such as IL-1β, IL-6, and TNF, are expressed. TNF is the first to be activated; its function is to recruit cells for regeneration, but if it remains active for too long, the inflammatory process can impair healing. It is at this point that CBD acts by decreasing pro-inflammatory cytokines (TNF) and aiding in the differentiation of bone marrow mesenchymal stem cells (BMSCs) into mature, functional bone cells under cytokine activation [27,31,32,33,34].

Thus, through integrated action on inflammatory, apoptotic, antioxidant, osteoregenerative and neuromodulatory pathways, cannabidiol proves to be a multifunctional molecule with high therapeutic potential, especially in contexts involving chronic pain, persistent inflammation and tissue regeneration processes.

## 4. Nerve Regeneration

Nervous tissue makes up the system responsible for receiving, sending, and transmitting internal and external signals in the human body: the Nervous System. The Nervous System, in turn, can be subdivided into the Central Nervous System (CNS) and the Peripheral Nervous System (PNS) and has neurons as its functional units [62]. These specialized cells can be classified according to their function and morphology [63].

In addition to differing in terms of function, structure, and location, neurons in the CNS and PNS respond differently to injuries they sustain [64,65]. Regarding the regenerative capacity, CNS neurons present great limitations when compared to PNS neurons, which, due to the presence of Schwann cells in the formation of the myelin sheath, have greater regenerative potential [65,66].

Thus, the nervous regenerative capacity of the CNS is primarily due to a mechanism called neural plasticity, which is defined as the brain’s physiological and structural adaptive ability in response to internal or external stimuli throughout life [67,68]. This property is of the utmost importance for maintaining the proper functioning of the CNS, as it is essential during neurodevelopment, being responsible for processes of learning and memory. It also has regenerative potential after brain injuries, playing a role in cognitive and motor recovery through the reorganization of neural networks following injuries associated with the CNS. CBD acts through the indirect activation of CB1 and TRPV1 receptors, regulating neurotransmitter release and stimulating signaling pathways such as BDNF/TrkB, PI3K/Akt, and ERK, thereby promoting the formation of new synapses and neuronal survival. Additionally, it preserves the integrity of neural networks through anti-inflammatory and antioxidant mechanisms, including NF-κB and ROS pathways, ultimately enhancing neuroplasticity under pathological conditions [68,69,70,71].

Therefore, endogenous factors, such as oxidative stress, and exogenous factors, such as trauma, can accelerate the process of cell membrane wear and neuronal death, leading to functional impairment [70]. This damage to nervous tissue can manifest in various ways, depending on the affected region or structure, and lead to the development of different types of neuropathologies that, when associated with environmental, genetic, and metabolic factors, can severely compromise the health and quality of life of the population [24].

Thus, cannabidiol promotes nerve regeneration primarily by modulating the endocannabinoid system (CB1, CB2) and receptors such as TRPV1, regulating neuronal excitability and neuropathic pain. Its anti-inflammatory action reduces cytokines such as TNF-α, IL-1β, and IL-6, while increasing mediators such as IL-10, creating a microenvironment conducive to repair. Furthermore, it activates intracellular pathways such as BDNF/TrkB, PI3K/Akt, and ERK, which promote neuroplasticity, cell survival, and the formation of new synapses, while its antioxidant properties neutralize reactive oxygen species. Thus, CBD acts in an integrated manner, protecting neurons from damage and stimulating their functional regeneration [72].

## 5. Cannabidiol Associated with Neurological Diseases

Regarding nervous tissue, CBD has been studied for its neuroregenerative potential and its association with the inhibition of disorders related to both the Central Nervous System and the Peripheral Nervous System [73]. The use of CBD-based medications as a complementary therapy is due to its neuroprotective potential through the activation of the endocannabinoid system, which, in recent studies, has shown efficacy in the treatment and regeneration of compromised nervous tissue [4,72].

**Figure 2:**
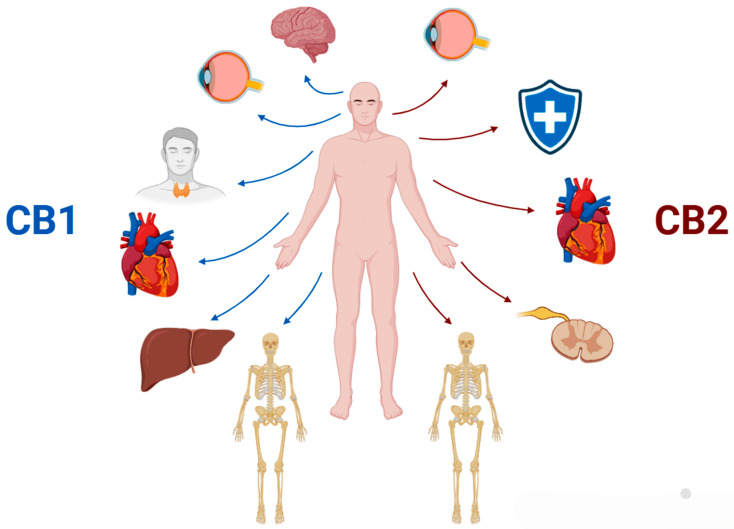
Representative image of the different body systems in which CB1 (cannabidiol receptor 1) and CB2 (cannabidiol receptor 2) receptors are present, playing a key role in activating the endocannabinoid system. CB1 is mainly present in the central nervous system and CB2 is mainly present in the peripheral nervous system and the immune system. Created by: Biorender.com.

Oxidative stress can be described as an imbalance in which there is an excess of free radicals over the antioxidants that neutralize them in a system, causing damage to cell membranes [74]. Due to the fact that the nervous system is composed of a tissue with high metabolic demand, the accumulation of free radicals from reactive oxygen species (ROS), such as H_2_O_2_ and O_2_^−^, primarily occurs as a byproduct of cellular respiration carried out by the mitochondria [4,74]. In this way, CBD plays an important role by reducing oxidative conditions in the environment through the inhibition of superoxide radical formation and modulating the activity of antioxidants, altering the redox balance of the system [4,75]. In addition to its action against oxidative stress, CBD-derived medications also have anti-inflammatory effects through the modulation of the CB1 and CB2 receptors of the ECS [76] (Figure 2).

Although CBD does not directly activate the ECS receptors, it can promote an increase in the expression of CB1 and CB2 receptors, which play a key role in regulating the immune response. Their activation reduces the production of pro-inflammatory cytokines such as TNF-α, IL-6, and IL-1β [4,77]. In this way, CBD works by preventing the exacerbation of inflammation in cells and tissues, thereby preventing further damage to nervous tissues adjacent to compromised structures.

### 5.1. Central Nervous System (CNS)

The Central Nervous System has the physiological role of processing information, coordinating activities, and regulating bodily functions [62]. Given the above, it is important to understand the etiology of the main dysfunctions that affect the CNS in order to comprehend the significance of CBD’s anti-inflammatory and antioxidant action in inhibiting the progression of disorders affecting this system [4,77].

#### 5.1.1. Epilepsy and Seizures

Epilepsy is a chronic neurological disorder caused by an excess of excitatory neurotransmitters (glutamate) over inhibitory ones (GABA), leading to abnormal, excessive, and synchronized electrical discharges in the brain. It is these uncontrolled electrical discharges that cause seizures, and in the case of recurrent episodes, changes in synaptic plasticity occur, leading the cells to emit irregular impulses [78].

Epileptic seizures increase brain metabolism, excessively releasing reactive oxygen species, which leads to oxidative stress, accelerating the degradation of nerve cells. In addition, pro-inflammatory cytokines are released, causing the brain to enter an inflammatory state, which also compromises the cellular function of neurons [79]. Recent studies conducted with mice have indicated the effectiveness of CBD’s neuroprotective properties in its anticonvulsant effects [80].

Recent studies conducted on mice have indicated the effectiveness of CBD’s neuroprotective properties in its anticonvulsant effects. This was observed through the study of the metabolic pathways by which it acts, revealing a reduction in in vivo neurodegeneration and neuronal death in cell cultures [80].

#### 5.1.2. Main Neurodegenerative Diseases

##### Alzheimer

Alzheimer’s disease (AD) develops from the deposition of the β-amyloid peptide (Aβ) between neurons, leading to the formation of senile plaques that hinder neuronal communication [81,82]. In addition, the hyperphosphorylation of the Tau protein, responsible for stabilizing neuronal microtubules, is another characteristic of AD, leading to the formation of neurofibrillary tangles and disrupting cellular transport [30,83].

Both processes lead the brain to a state of chronic inflammation, which, when combined with oxidative stress, causes the degradation and death of neurons, primarily in the hippocampus, the main center of memory and an important component of the limbic system [84].

CBD exerts neuroprotective effects in Alzheimer’s disease (AD) through multiple mechanisms. In addition to modulating neuroinflammation and exerting antioxidant effects by neutralizing free radicals, CBD reduces β-amyloid protein deposition via modulation of the PPARγ pathway and inhibits tau protein hyperphosphorylation through the regulation of GSK-3β kinase. These actions contribute to mitigating the potential damage of the disease on the patient’s nervous system [85].

Furthermore, research suggests that combining CBD with synergistic substances, such as vitamin B12, may enhance the beneficial effects of cannabidiol on the nervous system, even promoting hippocampal neurogenesis [86]. Despite the lack of studies in humans, research conducted on mice affected by the accumulation of Tau and beta-amyloid proteins, as well as oxidative stress, has indicated an extremely beneficial potential of CBD for Alzheimer’s disease. The studies showed improvements in learning and memory in the group treated with the substance compared to the control group (which did not receive CBD [84].

##### Parkinson

In Parkinson’s disease (PD), there is abnormal deposition of the α-synuclein protein, leading to the formation of protein aggregates called Lewy bodies, which, when combined with oxidative stress, damage the cell membranes of neurons, causing dysfunction and cell death [87,88,89].

The neurons affected by PD are located in the substantia nigra of the brain, a region of the midbrain responsible for dopamine production. These neurons can undergo oxidative stress triggered by inflammation caused by microglial hyperactivation, which leads to an excess production of pro-inflammatory cytokines such as tumor necrosis factor-α (TNF-α), interleukin 1 beta (IL-1β), and interleukin 6 (IL-6) [88,90]

The dopamine deficiency caused by these processes affects the communication of neurons in the basal ganglia, an important structure responsible for controlling movement, which leads to the primary symptoms associated with PD: motor coordination loss and resting tremors [14,86,89,91].

**Figure 3:**
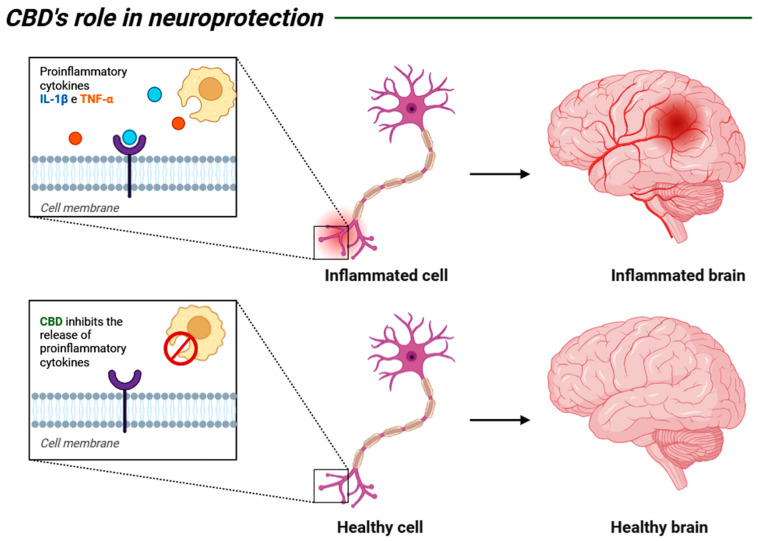
Schematic representation of the neuroprotective mechanism of CBD through its anti-inflammatory potential, which, by interrupting the release of pro-inflammatory cytokines such as TNF-α and IL-1β, inhibits the inflammatory response that may compromise nerve function and regeneration. Created by: Biorender.com.

Recent research shows a growing interest in associating CBD with the elderly population, as promising results from in vitro and in vivo studies have indicated the substance’s effectiveness in Parkinson’s disease. CBD has been shown to alleviate the motor disturbances characteristic of the disease, as well as reduce inflammatory markers, thereby decreasing neuronal damage caused by inflammation [90] (Figure 3).

##### Multiple Sclerosis

Multiple sclerosis (MS) is an autoimmune inflammatory disease in which the immune system attacks the multi-layered structure of the myelin sheath, causing inflammation and degradation of its plasma membrane and also promoting axonal loss [84,92,93,94]. This process of demyelination results in the formation of lesions and sclerotic plaques in the CNS, which impair neuronal communication, incorrectly dispersing the signals sent and received by the brain, affecting cognition, motor function, and sensitivity [94,95].

Thus, studies linking substances derived from *Cannabis sativa*, particularly CBD associated with THC, to multiple sclerosis aim to analyze the effects these compounds induce on the disease. Although studies in rodents have shown promising results, there is still a gap in experimental research in humans, making it difficult to determine the effects of cannabinoids on the disease in our species [94] (Table 1).

### 5.2. Peripheral Nervous System (PNS)

The PNS is responsible for the important function of mediating the transmission of pain stimuli received in the body’s periphery to the CNS, where these stimuli will be processed [97]. Pain, in turn, is an uncomfortable stimulus that can be classified according to various factors, including its intensity, origin, and duration [98]. Therefore, injuries caused by trauma, viral infections, autoimmune diseases, among other factors, can directly interfere with perception and adequate communication between the PNS and the CNS [97,99].

#### 5.2.1. Neuropathic Pain

Neuropathic pain is a disorder that affects the neurons of the PNS and was defined by the International Association for the Study of Pain (IASP) as pain resulting from trauma or pathology affecting the somatosensory nervous system [9,98]. As reported in a recent study conducted on rodents, CBD has an effect on the modulation of neuropathic pain by attenuating neuroinflammation through the endocannabinoid system, preventing the release of inflammatory substances onto the PNS [100]. CBD acts by blocking sodium channels, such as Nav1.7, as well as pain receptors TRPV1 and TRPA1, thereby attenuating post-injury neural hyperexcitability [101].

Furthermore, it is important to observe the effects produced by different routes of CBD administration in the treatment of various conditions, such as neuropathic pain. Drawing a parallel between the traditional administration methods of the substance, in vivo research has demonstrated the effectiveness of systemic CBD treatment on the sensory aspects of neuropathic pain induced in rodents. In contrast, the administration of cannabinoid products via oral or inhalation routes shows a significant lack of evidence regarding their effectiveness in treating neuropathic pain [102].

#### 5.2.2. Chronic Pain

**Figure 4:**
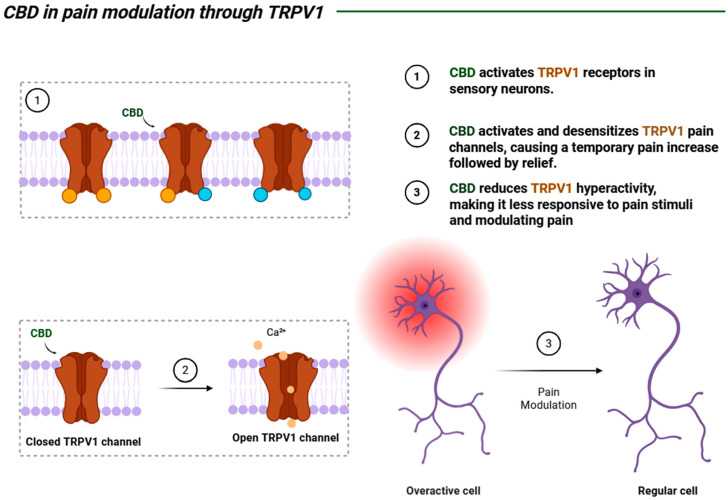
A schematic representation of the direct interaction between CBD and the TRPV1 receptor (a transmembrane ion channel) embedded in the neuronal plasma membrane, which is one of the processes through which CBD can modulate pain in the peripheral nervous system (PNS). Created by: Biorender.com.

Chronic pain is a debilitating challenge for countless people worldwide, characterized by a continuous or recurrent perception of an unpleasant stimulus that lasts for a period of at least 3 to 6 months [9,14,103]. This abnormal pain originates from an inflammatory process, in which tissue damage leads to hypersensitization of the injured area, and it can be modulated by CBD through the ECS, by interacting with the CB1 receptor and pain channels, such as TRPV1, reducing the hyperactivity of the affected nerve and its interaction with CB2 in controlling local inflammation [102,103,104,105] (Figure 4).

Thus, studies conducted using CBD combined with THC in elderly patients have indicated the effectiveness of these substances in pain relief by modulating pain perception in these patients, presenting a promising future alternative to the use of opioid medications [14]. Furthermore, it is important to study the mechanisms mediating the action of this substance to understand its clinical applicability and underlying processes. This was demonstrated in a study that analyzed the pathways involved in pain modulation and neuroinflammation attenuation in Wistar rats [100] (Table 2).

## 6. Bone Regeneration

Bone is an extremely important tissue for the body, considered a vital organ for the regulation of physiological resources, it is responsible for providing structural support, protecting other organs, and contributing to bodily movements [108]. This tissue has a unique composition that depends on systemic and local factors to remain healthy, in other words, the homeostasis of the skeletal system requires balanced hormones and growth factors for bone remodeling to occur—an activity between osteoblasts and osteoclasts that is essential for bone regeneration and repair [109,110].

This osteoregenerative process encompasses several factors, such as an inflammatory response triggered by cytokines, the formation of new blood vessels, tissue interaction, and the interplay of multiple cells [111]. By observing this fact, it is possible to understand why various diseases and medical interventions in bone injury repair are influenced, considering intrinsic and extrinsic factors such as advanced age, quality of life, type of trauma, and chronic diseases negatively affect the bone regeneration process, often requiring external intervention through medications and even surgeries [108,112,113].

**Figure 5:**
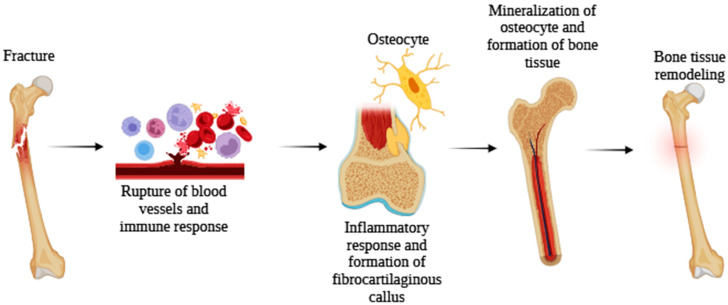
Illustrative diagram of the bone regeneration process, consisting of the formation of a hematoma resulting from the rupture of blood vessels at the time of injury. This fact stimulates the immune system’s response, which attracts bone progenitors. Thus, the chondrocytes form a fibrocartilaginous tissue that subsequently hypertrophies and forms new bone tissue, which will undergo several remodelings to adapt to the new tissue. Created by: Biorender.com.

Bone regeneration generally occurs in this way: at the time of injury, some vessels rupture, releasing cells and plasma mediators. In the first stage, called the initiation phase, the precursor cells become osteoclasts, which synthesize enzymes that perform bone reabsorption. These cells are activated by specific signaling molecules (formation of fibrocartilaginous callus). After this, during the inversion phase, the action of osteoclasts is interrupted by molecules that halt the process, leading to the apoptosis of these cells [114]. Finally, the last phase, characterized as the terminal phase, involves the synthesis of osteoblasts, which are differentiated through bone morphogenetic proteins (BMPs). These proteins are responsible for the production of the bone matrix, which will later undergo mineralization through calcium ions. Thus, after all these stages, the complex process of bone regeneration can be considered complete. However, it may take months or even years, depending on the severity and extent of the injury, as well as the overall health of the individual with the injury [55] (Figure 5).

Thus, in bone regeneration, CBD acts from the inflammatory phase to final remodeling, modulating pro-inflammatory cytokines (TNF-α, IL-1β, IL-6) and favoring the differentiation of mesenchymal stem cells into osteoblasts. This effect is mediated primarily by the activation of the CB2 receptor, the p38 MAPK pathway, and WNT/β-catenin signaling, which stimulate osteogenesis and mineralization. At the same time, CBD reduces excessive osteoclastic activity, promoting a balanced remodeling process and increasing the mechanical strength of the formed bone. Thus, it combines anti-inflammatory and osteoinductive effects, constituting a key modulator in the bone repair and consolidation process [115].

## 7. Cannabidiol and Bone Disease

With the advancement of scientific research, the prescription of cannabidiol and its derivatives has been increasing in the global market, with growing interest in the therapeutic use of the substance for the treatment of numerous diseases [116,117]. However, alongside research demonstrating the efficacy of the product, there are several regulatory obstacles and misconceptions related to the psychotropic effects of cannabis as opposed to the pharmacological effects of phytocannabinoids [118,119]. In light of the above, in order to avoid erroneous interpretations about the effects of cannabidiol, it is important to understand how the substance functions in the body, with a focus on its regenerative processes [40].

**Figure 6:**
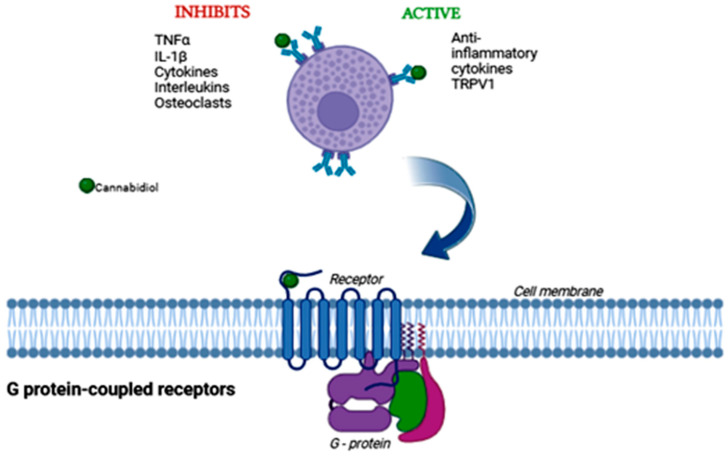
Illustrative diagram of the process of activation of protein G by cannabidiol. Protein G is a membrane receptor, so CBD needs to reach the cell membrane to perform its effect. This can be activation or inhibition, as described in the image. Created by: Biorender.com.

Therefore, the mechanism of cannabidiol begins with the binding to several receptors coupled with G proteins, ionotropic receptors, enzymes, transporters and nuclear factors. It is important to emphasize the importance of the G protein complex for CBD activation, as it is a membrane receptor, cannabidiol needs to reach the cell and connect to its receptor, so that the desired effect occurs, such as: activation of anti-inflammatory effects and inhibition of osteoclastic activity [9]. In the case of bone fractures, CBD, during the initial phase of regeneration, promotes stimuli that increase periosteal bone progenitors at the site of the hematoma, meaning it helps in bone neoformation. It also accelerates the mineralization of the fibrocartilaginous callus in the period following the trauma, increasing the proliferation of bone cells and the quality of the forming bone [23] (Figure 6).

Moreover, CBD not only acts in regeneration but also encompasses the entire recovery process of an injury, including pain management. Recent studies show that the use of non-steroidal anti-inflammatory drugs and opioids brings harmful effects, the overall recovery, as it impedes the inflammatory process of an injury, thus hindering healthy bone remodeling, as well as causing bone loss, fractures, and addiction [23,120]. While cannabidiol, when used correctly, can aid in bone regeneration and provide pain relief throughout the healing process of an injury [115]. As reported in a study involving femur fractures in rats, CBD reduced sublesional trabecular bone loss and improved the mechanical properties of the newly formed bone [121].

Thus, in a clinical setting, the most common form of bone regeneration occurs from fractures. Under normal physiological conditions, bone tissue has the capacity to regenerate autonomously. However, in cases of large-scale injuries—such as trauma, infections, tumors, or congenital anomalies—the regeneration process becomes more complex and often insufficient for complete recovery. Furthermore, diseases such as osteoporosis can delay and further compromise this repair process [122].

Considering these situations in which bone regeneration is deficient or non-existent, different therapeutic approaches have been developed, with autologous bone grafts or allografts currently considered the gold standard. However, these techniques have significant limitations, such as the risk of surgical complications, immunological rejection, and a long recovery period [122].

In this context, cannabidiol has emerged as a potential adjunct to conventional treatments, acting both to control pain and support the osteoregenerative process. Although further studies are needed to confirm whether CBD can effectively replace grafts as the primary treatment for extensive bone lesions—especially given the scarcity of specific research in this area—preliminary evidence indicates that CBD has promising bone regeneration properties, constituting a potential future therapeutic approach [23].

For a more in-depth assessment of the effects of CBD on bone regeneration, it is important to analyze the results of recent studies. To this end, a table was created that compares the six main studies on bone regeneration and the use of CBD (Table 3).

An analysis of Table 3 highlights the significant growth in scientific interest in the use of cannabidiol in bone regeneration over the last decade. The studies presented, mostly preclinical (in vitro and in vivo), demonstrate that CBD acts at different stages of the osteoregenerative process, from modulating initial inflammation to osteogenic differentiation and tissue mineralization. This diversity of experimental models, including dental pulp stem cells, bone marrow mesenchymal cells, and animal fracture models, reinforces the compound’s versatility in different biological and pathological contexts.

The studies presented in the table highlighted CBD as a possible ally in the treatment of bone injuries and diseases, highlighting its anti-inflammatory and antioxidant properties as the main factors responsible for the positive results observed [5,23,40,115,119]. An experiment in vitro study demonstrated that CBD, when applied to dental pulp stem cells, stimulated a significant osteogenic potential, through the regulation of the WNT6 gene, which is part of the WNT signaling pathway associated with osteogenesis [5]. Similarly, another experimental study used dental pulp stem cells in basal and inflammatory environments and observed that cannabidiol was able to promote an osteogenic effect through the modulation of pro-inflammatory cytokines. These two studies, although with some differences in their experimental models, present similar and favorable conclusions regarding the effects of CBD on dental pulp stem cells, suggesting its potential for dental treatments of bone injuries and diseases [40].

A clinical report of postmenopausal women diagnosed with osteopenia demonstrated favorable results after 12 weeks of oral CBD use. Assessment using serum bone resorption markers indicated positive bone remodeling. These findings reinforce CBD’s potential as an ally in the treatment of bone diseases [123].

A 2020 experimental study presented an interesting finding when investigating the osteogenic differentiation potential of combined cannabidiol and vitamin D3 treatment in dental mesenchymal stem cells [119]. This result is noteworthy because it suggests the possibility of combined therapeutic strategies for bone injuries, in which CBD could be more effective when combined with other substances. It is known, for example, that the combined administration of CBD and tetrahydrocannabinol (THC) increases plasma THC concentrations but promotes an antagonistic effect on analgesia, demonstrating that CBD, when combined with certain compounds, can exhibit synergistic or even antagonistic interactions, especially with molecules of similar origin [40,124].

Despite its potential, this hypothesis still requires further investigation, making it impossible to draw definitive conclusions about the true efficacy of combined therapies involving CBD in the treatment of degenerative bone diseases. Therefore, it is essential to conduct further studies evaluating the effects of CBD alone and in combination with other drugs or bioactive agents [124]. Supplementary Material (Table S1) was prepared to present the main clinical trials on CBD applications, including both the dosages used and the results obtained [125,126,127,128,129,130,131,132].

In this context, this review makes a significant contribution by bringing together and critically analyzing the key findings on the role of CBD in bone regeneration. By integrating recent evidence and comparing it with mechanisms already described in the literature, the work offers a comprehensive and up-to-date overview of the topic. This approach is of great importance because it clarifies existing gaps, such as the need for human clinical trials, and at the same time highlights the opportunities that CBD represents as a less invasive and potentially more effective alternative to traditional therapies, such as bone grafts. Thus, by discussing the consolidation of CBD as an emerging agent in osteoregeneration, this review reinforces the importance of expanding translational and clinical research, contributing to transforming laboratory findings into concrete medical applications.

## 8. Conclusions

This review seeks to consolidate and critically evaluate the most relevant studies published in recent decades concerning the effects of cannabidiol (CBD) on tissue regeneration within both the central and peripheral nervous systems, as well as in bone tissue. By examining preclinical and clinical evidence, the review aims to elucidate the potential mechanisms through which CBD may contribute to neuroregeneration and osteogenesis, highlighting its therapeutic promise in the context of neurodegenerative diseases, nerve injury, and bone disorders.

The mechanisms underlying CBD’s therapeutic effects involve modulation of the endocannabinoid system, downregulation of pro-inflammatory cytokines, upregulation of anti-inflammatory mediators, and activation of intracellular signaling cascades that support both neuroplasticity and osteogenic differentiation. These converging actions contribute not only to cellular protection but also to the stimulation of critical tissue repair processes, such as bone matrix mineralization and synaptogenesis. Collectively, these effects underscore the potential of CBD as a regenerative agent in pathological conditions related to aging, trauma, and neurodegenerative or musculoskeletal disorders. This review offers a comprehensive synthesis of current findings, emphasizing the innovative potential of cannabidiol (CBD) as a minimally invasive and multifunctional therapeutic strategy for the regeneration of nerve and bone tissues. By integrating recent preclinical and clinical evidence, and identifying critical gaps in the literature, this work establishes a robust foundation for future investigations. Furthermore, it reinforces the relevance of CBD as a promising agent in promoting tissue repair and functional recovery, particularly in contexts associated with aging, trauma, and degenerative disorders, thereby contributing to improved health outcomes and quality of life.

## Figures and Tables

**Table 1:** Relevant studies on the use of CBD on CNS dysfunctions.

Article	Kind Study	Objective	CNS	CBD	Mainly Outcomes
Taylor et al., 2018 [42]	Clinical Study	This investigation examined the safety, tolerability and pharmacokinetics of orally administered CBD in healthy volunteers.	Neuroprotection.	CBD	Results indicate that oral doses of CBD up to 6000 mg are well tolerated and are swiftly and extensively metabolized into the active compound 7-OH-CBD.
Lima et al., 2020 [80]	Experimental In vitro and animal study	Investigate the mediation pathways of the anticonvulsant and neuroprotective effects of CBD through an experimental study.	Neuroregeneration and neuroprotection.	CBD	CBD induced an anticonvulsant effect, in addition to reducing neurodegeneration in vivo and neuronal death in cell cultures.
Troján et al., 2023 [84]	Review	Conduct a review on the beneficial effects of CBD use and explore its potential applications in clinical practice.	Neuroprotections and Alzheimer.	CBD	More clinical studies are needed to build stronger evidence; however, in most cases, CBD induced beneficial effects on Alzheimer’s and in the treatment of other conditions in elderly individuals.
Costa et al., 2022 [90]	Review	Explore the role of CBD as an adjunctive therapy in the treatment of PD and dementia in the elderly.	Neuroprotection and Parkinson’s Disease.	CBD	Improvement of sleep quality, anxiety, and tremors caused by it, indicating an improvement in the quality of life of patients with PD.
Nicolau et al., 2023 [96]	Clinical Study	To deliver an extensive analysis of nabiximols spray (CBD+THC) therapy for spasticity in MS across two clinical studies.	Neuroprotection and Multiple Sclerosis.	CBD and THC	The sustained spasticity reduction observed with nabiximols over 12 weeks underscores its potential as a clinically meaningful intervention, as reflect in spasticity scores, spasm counts and muscle assessments.

List of Abbreviations Appearing in the Table: CDB—cannabidiol; CNS—central nervous system; PD—Parkinson’s disease; THC—tetrahydrocannabinol; MS—multiple sclerosis; 7-OH-CBD-7—Hydroxycannabidiol.

**Table 2:** Relevant studies on the use of CBD on PNS dysfunctions.

Article	Kind Study	Objective	PNS	CBD	Mainly Outcomes
Silva-Carsoso et al., 2023 [100]	Experimental Animal study	Evaluate whether sub chronic CBD treatment could be associated with conditioned pain reversal through a study in rodents.	Pain modulation and Neuroprotection.	CBD	Systemic treatment with CBD had a positive effect on the modulation of pain or the emotional behavior associated with pain, as well as reversing the expression of proteins that may hinder neuronal regeneration and synaptic plasticity.
Capano et al., 2019 [10]	Clinical Study	Examine the effects of full-spectrum hemp-derived CBD on opioid consumption and quality of life outcomes in individuals with chronic pain.	Pain Modulation and Chronic Pain.	CBD and opioid	The use of CBD may contribute to a reduction in opioid intake and provide improvements in chronic pain management as well as sleep quality among individuals dependent on opioid therapy.
Chaves et al., 2020 [106]	Clinical Study	To investigate the potential therapeutic benefits of a THC-rich, CBD-containing cannabis oil on pain modulation and quality of life among individual with fibromyalgia.	Pain Modulation and Chronic Pain.	CBD and THC	Treatment with cannabis derivatives was associated with reduced pain scores relative to placebo, indicating their potential as an affordable and well-tolerated option for improving symptoms and quality of life in chronic pain patients.
Macêdo-Souza et al., 2023 [105]	Experimental Animal study	Investigate the effects of chronic systemic CBD treatment on pain alteration in rats subjected to neuropathic pain.	Pain Modulation.	CBD	The treatment results indicated the efficacy of systemic CBD treatment on the sensory aspects of chronic neuropathic pain.
De Vita et al., 2021 [107]	Clinical Study	This study aimed to experimentally assess the impact of CBD and the influence of expectancy on pain responses in humans.	Pain Modulation and Analgesia.	CBD	The pilot findings suggest that CBD and analgesic expectations independently and jointly reduce pain discomfort, underscoring the need for further research on the mechanisms driving CBD analgesia.

List of Abbreviations Appearing in the Table: CBD—cannabidiol; THC—tetrahydrocannabinol.

**Table 3:** Relationship between bone regeneration and use of Cannabidiol.

Article	Kind Study	Objective	Bone Regeneration	CBD	Main Outcomes
Khajuria et al., 2023 [23]	Experimental Animal study	To investigate the impact of CBD and CBG on the different stages of healing of a bone injury.	Bone fracture in mouse	CBD and CBG	CBD and CBG intensify the supervision of bone cells, this fact causes an increase in bone and mineral volume, boosting the mineralization of fibrocartilaginous heat.
Liu et al., 2024 [5]	Experimental In situ	To develop DPSCs based osteogenic microspheroids for the treatment of bone regeneration using CBD as osteoinduction.	Dental pulp stem cells	CBD	Due to the upregulation of WN6, DPSCs treated with CBD showed satisfactory bone regenerative potential.
Petrescu et al., 2020 [119]	Experimental In vitro	To determine a differentiation protocol using CBD and Vit. D3 for osteogenic differentiation of mesenchymal stem cells derived from dental tissue.	Osteogenic Differentiation	CBD and Vit. D3	CBD and vitamin D3 enhance osteogenic differentiation potential in dental tissue derived mesenchymal stem cells.
Li et al., 2022 [115]	Experimental In vitro	To investigate the efficiency of CBD in the osteogenic differentiation of BMSCs in the inflammatory microenvironment.	Bone marrow mesenchymal stem cells	CBD	CBD performed osteogenic differentiation of BMSCs by CB2/p38 MAPK signaling in the inflammatory microenvironment.
Yu et al., 2023 [40]	Experimental In vitro	To analyze the effect of CBD on the minority, migration and mineralization of DPSCs.	Dental Pulp Stem Cells	CBD	The application of CBD in DPSCs resulted in the osteogenic differentiation of the cells in question and inhibited the action of pro-inflammatory cytokines.
Kulpa et al., 2023 [123]	Clinical Study	To explore the effects of oral CBD administration on bone remodeling in two postmenopausal women with osteopenia	bone remodeling	CBD	CBD was well tolerated after 12 weeks of twice-daily oral administration and was associated with a reduction in bone turnover markers

List of Abbreviations Appearing in the Table: CBD—cannabidiol; CBG—cannabigerol; Vit.D3—vitamin D3; BMSCs—bone marrow mesenchymal stem cells; CB2—cannabinoid receptor 2; p38—phosphorylated 38; MAPK—mitogen activated protein kinase; DPSCs—dental pulp stem cell.

## Data Availability

Not applicable.

## Supplementary Materials

The following supporting information can be downloaded at: https://www.mdpi.com/article/10.3390/biomedicines13102490/s1, Table S1: Clinical studies using CBD.
