# Supplementary material for: Exploring Cannabidiol’s Role in Regenerative Medicine: Focus on Neural and Skeletal Tissues
Source: Biomedicines. 2025 Oct 13;13(10):2490. doi: 10.3390/biomedicines13102490 (PMC12562014; doi:10.3390/biomedicines13102490)
Supplement: Supplementary file 1 [file biomedicines-13-02490-s001.zip › biomedicines-3855714-supplementary.pdf]

Table S1. Clinical studies using CBD

Article	Kind study	Objetive	application of CBD	Mainly Outcomes
Kulpa et al., 2024 [125]	Phase 1 Clinical trials, double-blind, placebo-controlled.	To explore the effects of oral CBD and THC on normal bone homeostasis by assessing markers of bone resorption and formation.	Bone resorption and bone formation.	Short-term treatment with CBD or THC resulted in attenuation of a marker of bone resorption.
Brazeau et al., 2025 [126]	Randomized, triple-blind, placebo-controlled clinical trial.	To evaluate the effect of CBD treatment on pain, inflammation and self-reported well-being after a fracture injury.	CBD for the treatment of pain and inflammation after a long bone fracture.	The study has not yet been completed; it started in January 2025 and is scheduled to end in January 2027.
O'Brien et al., 2022 [127]	Randomized, double-blind, placebo-controlled trial.	To analyze the efficacy, safety, and tolerability of transdermally administered cannabidiol in adults.	CBD for the treatment of drug-resistant focal epilepsy in patients.	Transdermal cannabidiol doses were well tolerated and safe. However, a significant difference in efficacy was observed between cannabidiol and placebo; higher doses may be necessary for the desired effect.
Devinsky et al., 2017 [128]	Double-blind, placebo-controlled clinical trial.	To study the use of cannabidiol for the treatment of drug-resistant seizures in Dravet syndrome.	CBD for the treatment of seizures.	Cannabidiol resulted in a greater reduction in seizure frequency but was associated with higher rates of adverse events such as diarrhea, vomiting, and fatigue.
Zheng et al., 2023 [129]	Randomized, double-blind, placebo-controlled clinical study.	Compare the effects of 4 weeks of CBD administration with placebo in individuals with IG or DM.	Application of cannabinoids to reduce nausea and pain related to inflammation.	CBD reduced pain scores through the reversal of hypersensitivity and intrinsic inflammatory pathogenesis.
Chrepa et al., 2023 [130]	Investigator-initiated	Evaluate the safety and analgesic effect	Application of CBD as an	Demonstrated the effectiveness of the analgesic

	triple-arm, phase IIA, randomized placebo-controlled trial with double masking.	of CBD in the treatment of emergency dental pain.	alternative to NSAIDs and opioids.	effect of CBD in the treatment of acute inflammatory pain.
Umpreecha et al., 2023 [131]	Randomized parllel double-controlled trial design.	Evaluate the clinical safety and effectiveness of topical CBD in the treatment of RAU.	Application of 0.1% topical CBD as a substitute for topical steroids.	CBD exerted anti-inflammatory effects, accelerated healing, and promoted the reduction of the ulcerated area.
Spinella et al., 2021 [132]	Randomized crossover study	Evaluate the influence of CBD expectations on stress, anxiety, and mood	The expectation of CBD administration induced a positive response from the body regarding stress and anxiety.	The expectation of CBD application generated positive subjective and physiological responses reported by the study patients.

List of Abbreviations Appearing in the Table: CBD-cannabidiol; THC-delta-9-tetrahydrocannabinol; idiopathic gastroparesis; DM-diabetic gastroparesis; NSAIDs-nonsteroidal anti-inflammatory drugs; RAU-recurrent aphto us ulcer.
